# Normocalcemic Hyperparathyroidism: Study of its Prevalence and Natural History

**DOI:** 10.1210/clinem/dgaa084

**Published:** 2020-02-19

**Authors:** Marian Schini, Richard M Jacques, Eleanor Oakes, Nicola F A Peel, Jennifer S Walsh, Richard Eastell

**Affiliations:** 1 Department of Oncology and Metabolism, University of Sheffield, Sheffield, UK; 2 School of Health and Related Research, University of Sheffield, Sheffield, UK; 3 Sheffield Teaching Hospitals National Health Service Foundation Trust, Sheffield, UK

**Keywords:** normocalcemic hyperparathyroidism, prevalence, natural history, epidemiology, primary hyperparathyroidism

## Abstract

**Context:**

Normocalcemic hyperparathyroidism (NPHPT) is characterized by persistently normal calcium levels and elevated parathyroid hormone (PTH) values, after excluding other causes of secondary hyperparathyroidism. The prevalence of the disease varies greatly and the data on the natural history of this disease are sparse and inconclusive.

**Objectives:**

The objectives of this study are to describe the prevalence of NPHPT and its natural history in a referral population and to compare the variability of serum calcium with a group of patients with primary hyperparathyroidism (PHPT).

**Design:**

A retrospective study was conducted over 5 years.

**Setting:**

The setting for this study was a metabolic bone referral center.

**Patients:**

A total of 6280 patients were referred for a bone mineral density measurement (BMD).

**Main Outcome Measures:**

The prevalence and natural history of NPHPT and variability of calcium were the main outcome measures.

**Results:**

We identified NPHPT patients using data from the day of the BMD measurement. We excluded patients with low estimated glomerular filtration rate (eGFR) or vitamin D, or with no measurements available. Based on the evaluation of their medical files, we identified 11 patients with NPHPT (prevalence 0.18%). Only 4 patients had consistent normocalcemia throughout their follow-up, with only 2 also having consistently high PTH. None had consistently normal eGFR or vitamin D.

Intermittent hypercalcemia was present in 7 of the 11 NPHPT patients. The mean adjusted calcium was found to be significantly lower in the NPHPT group compared with the PHPT group but higher than the control group. PTH was similar for NPHPT and PHPT. These 2 groups had similar variability in serum calcium.

**Conclusions:**

NPHPT patients often have episodes of hypercalcemia. We believe that NPHPT is a mild form of PHPT.

Normocalcemic hyperparathyroidism (NPHPT) is a disorder of calcium metabolism that, despite being mentioned in the literature for several years, was officially defined only in 2009, during the Third International Workshop on Asymptomatic Primary Hyperparathyroidism (PHPT) (1). According to the latest guidelines, persistently normal calcium levels (total and ionized) and consistently elevated parathyroid hormone (PTH) values characterize NPHPT. The panel suggested that normal calcium has to be confirmed on several occasions and that an elevated PTH measurement should be confirmed on at least 2 consecutive measurements. Other causes of secondary hyperparathyroidism have to be excluded; these include medications known to affect PTH levels (diuretics, lithium, denosumab, bisphosphonates, anticonvulsants, and phosphorus), low vitamin D, chronic kidney disease (estimated glomerular filtration rate [eGFR] < 60 mL/min/1.73 m^2^), renal calcium loss (hypercalciuria) and diseases of the gastrointestinal tract known to affect calcium absorption (celiac disease, inflammatory bowel disease, and bariatric surgery) (1-3).

NPHPT is usually encountered during the laboratory evaluation of osteoporosis and has been associated with consequences known to affect patients with PHPT (accelerated bone loss, kidney stones, higher levels of blood pressure, and higher preexisting glucose intolerance). However, there is no clear evidence for any such associations (4, 5).

The prevalence of NPHPT in the literature varies considerably, and it is reported to be between 0.1% and 8.9% (5). The reasons for this variation are that different definitions and methodologies were used in the reported studies; not all causes of secondary hyperparathyroidism were excluded, and some studies did not test the persistence of high PTH and normal calcium before evaluating the prevalence; thus the results were probably overestimated.

The data on the natural history of this disease are also sparse and inconclusive. Some studies show progression to hypercalcemia, but others do not. The way that many studies identify NPHPT patients is by testing calcium and PTH at baseline, and retesting after some time to check whether they progressed to hypercalcemia. The variability of calcium in NPHPT during the follow-up period has been poorly described. One study has described the variability as similar to the normal population, whereas patients with PHPT have been described to have more considerable variability in calcium levels (6). However, there are not enough data to draw any conclusions.

According to the proceedings of the Fourth International Workshop on Asymptomatic Primary Hyperparathyroidism, NPHPT remains incompletely described, especially regarding its epidemiology, natural history, and management (4). The criteria are not clear concerning how many measurements are needed to establish a diagnosis, which makes the matter complicated for the clinician and confusing for the patient. This study aims to identify the prevalence of NPHPT and study the natural history of this disorder in more detail. It also aimed to compare the variability of calcium and PTH between PHPT and NPHPT patients.

## Methods

### Study population

This work was performed at the Metabolic Bone Centre (MBC) at Sheffield Teaching Hospitals National Health Service Foundation Trust (STH NHS FT) in the United Kingdom. Data from patients referred for a bone mineral density (BMD) measurement between January 14, 2013 and July 27, 2017 were retrospectively evaluated. Patients were included in the database if they had a laboratory evaluation, including calcium and PTH, performed within 28 days of their scan. In general, this department evaluated approximately 650 patients per month at the time of the study and a laboratory workup for secondary osteoporosis was performed in patients having any of the following findings: low BMD for age (Z score < –2.0), vertebral fracture, or unexplained bone loss since the previous scan. The day of the laboratory investigations was defined to be the index day. More results of calcium and PTH before and after the index day were retrieved from hospital records and were used to study the natural history of the disease. Only data collected after January 14, 2013 were evaluated because at that point, the laboratory changed the assay for serum calcium, and this would probably affect the accuracy of the results. The end of the follow-up period was July 31, 2018.

The analysis was considered a case note review project by Sheffield Teaching Hospitals and did not require a special ethical approval (project number STH20617).

### Biochemical measurements

Blood was drawn at any point of the day, so patients were not fasting. All the samples were analyzed in the Chemical Pathology Laboratory, STH NHS FT. Intact PTH (second generation) was measured using an immunoassay method by the Roche Cobas 8000 e602 (Roche Diagnostics GmbH). The interassay coefficient of variation (CV) measured in the laboratory is 2.2% to 3.2% at 34 ng/L, 1.6% to 1.7% at 94 ng/L, and 1.4% to 1.8% at 839 ng/L, and the reported reference interval is 15 to 65 ng/L (1.6-6.9 pmol/L).

Serum calcium was measured using a Roche/Hitachi Cobas 8000 e702 automated clinical chemistry analyzer (Roche). This method uses 5-nitro-5’-methyl-BAPTA reagent. The interassay CV as measured in the laboratory is 1.1% to 1.5% at 1.52 mmol/L and 0.6% to 1.1% at 3.07 mmol/L. Albumin measurement was performed using a Roche/Hitachi Cobas 8000 e702 analyzer (Roche). The interassay CV as measured by the laboratory is 1.5% to 2.4% at 33.9 g/L and 1.0% to 1.7% at 59.7 g/L. The albumin-adjusted calcium was used for this study and was calculated using the following equation based on data from the local laboratory. The method by which the laboratory established its equation was based on a protocol published by Barth et al (7).

$$\begin{array}{l} Adjusted\;Ca=Total\;Ca+[0.0172\left(43-Albumin\right)] \end{array}$$

The reference interval for adjusted calcium is 2.20 to 2.60 nmol/L (8.8-10.4 mg/dL).

25-hydroxyvitamin D (25(OH)D) was measured using the IDS-iSYS automated immunoassays (Immunodiagnostic Systems) until May 2013. The interassay CV reported by the manufacturer was 10.4% at 64.5 nmol/L. The assay then changed to competitive binding protein and was performed by Roche modular analytics Cobas E170 (Roche). The laboratory interassay CV for this assay is 6.5% to 9.9% at 48.2 nmol/L and 4.5% to 6.3% at 92.3 nmol/L. The Sheffield Teaching Hospitals laboratory was participating in the UK Vitamin D External Quality Assessment Scheme at the time of this study.

Creatinine was measured using a Roche/Hitachi Cobas c8000 e702 analyzer (Roche). The interassay CV for this assay was 2.7% to 6.1% at 55.7 μmol/L and 2.3% to 4.0% at 458 μmol/L. The equation used to calculate eGFR was the Modification of Diet in Renal Disease Study equation:

$$eGFR\left(\frac{mL}{min\times 1.73\;m^{2}}\right)=175\times {\left(\text{Serum }\;\text{ creatine}\right)}^{-1154}\times age^{-0.203}\times \left[0.742\;if\;female\right]\;\times \;[1.212\;if\;black]$$

This equation changed in August 2015; the Chronic Kidney Disease Epidemiology Collaboration (CKD-EPI) equation has been in use since then.

$$\begin{array}{ll} eGFR\left(\frac{mL}{min\times 1.73\;m^{2}}\right)= & 141\times \min{\left(\frac{Scr}{k},\;1\right)}^{\alpha }\\ & \times \max{\left(\frac{Scr}{k},\;1\right)}^{-1.209}\times 0.993^{age}\\ & \times 1.018\;\left[if\;female\right]\;\times \;1.159\;[if\;black] \end{array}$$

Scr is serum creatinine in mg/dL, κ is 0.7 for female and 0.9 for male patients, α is –0.329 for female and –0.411 for male patients, min indicates the minimum of Scr/κ or 1, and max indicates the maximum of Scr/κ or 1 (8).

Alkaline phosphatase was measured using a Roche/Hitachi Cobas c8000 e702 analyzer. The interassay CV for this assay was 2.4% at 92.8 IU/L and 1.7% at 224 IU/L. The same analyzer was used for the measurement of serum phosphate and the interassay CV was 1.4% at 1.23 mmol/L and 1.2% at 2.04 mmol/L.

BMD was performed using dual-energy X-ray absorptiometry (DXA) of the lumbar spine and the proximal femur in posteroanterior projection. At the time of the study, there were 3 Hologic DXA scanners within the center. The least significant change (LSC) used in the MBC was 4.5% both for the spine and the hip. There were 3 scanning rooms in the department and each room has a different scanner. Room A: Hologic Discovery A has been in use since 2010. Room B: Hologic QDR4500A was in use from 2011 until September 11, 2013. The scanner was then decommissioned and replaced by a Hologic Discovery SL. Room C: Hologic Delphi C was in use until November 3, 2014. The scanner was then decommissioned and replaced by a Hologic Horizon A. The different DXA scanners were cross-calibrated at installation.

## Statistical Analysis

Because calcium and PTH are not independent variables, a bivariate statistical approach was used to define the different categories of patients. Mahalanobis distance is a multidimensional generalization of the concept of measuring the number of SDs away an observation is from the mean of a distribution. It is a classical method of defining outliers in a multivariate point cloud and is defined for each point x_i_, by the following equation, where MD_i_: Mahalanobis distance, μ: arithmetic mean of the data set, and S: sample covariance matrix.

$$\begin{array}{l} MD_{i}^{2}={\left(x_{i}-\mu \right)}^{T}S^{-1}(x_{i}-\mu ) \end{array}$$

**Figure 1.**
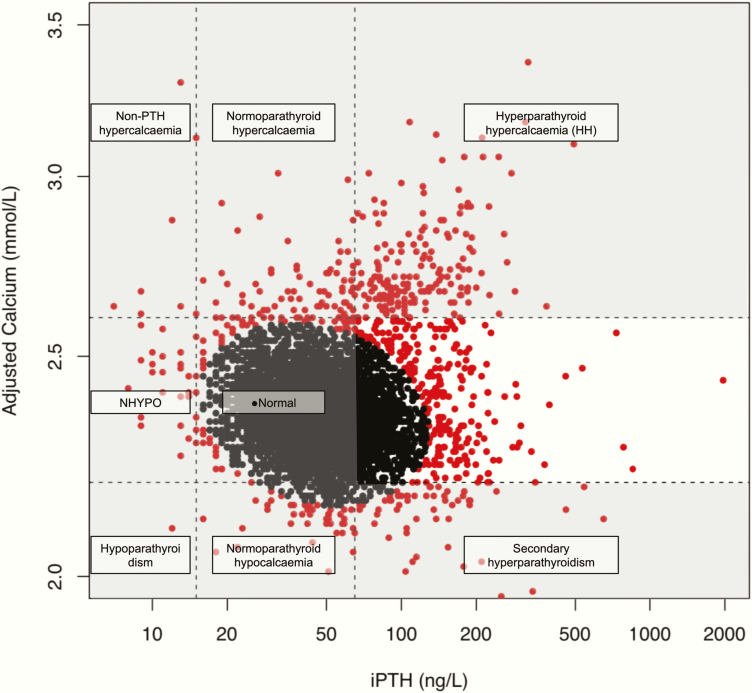
Data results from adjusted calcium and parathyroid hormone (PTH). The ellipse was formed using a statistical method (Mahalanobis distance) to identify “normal” individuals (black dots) and “abnormal” ones (red dots). The reference interval both of adjusted calcium and PTH (horizontal and vertical dashed lines, respectively), were used to identify patient categories. The definitions used are described in the “Methods” section. The white area includes patients with normal adjusted calcium and high PTH (n = 265); these were either potentially NPHPT patients (given that 25[OH]D ≥ 50 nmol/L and estimated glomerular filtration rate [eGFR] ≥ 60 mL/min/1.73 m^2^) or secondary hyperparathyroidism patients (given that 25[OH]D < 50 nmol/L and/or eGFR < 60 mL/min/1.73 m^2^). NPHPT, normocalcemic hyperparathyroidism; NHYPO, normocalcemic hypoparathyroidism.

The distance MD_i_ gives the distance of point x_i_ from the center of the cluster of points, taking into account the shape of the cluster. Observations are considered outliers if MD^2^ > Χ ^2^_2;0.975_ = 7.378 (97.5th percentile of chi-squared distribution with 2 degrees of freedom) (9, 10). The correlation analysis of adjusted calcium and PTH was performed based on a log10 transformation of the 2 variables. A similar approach has been used in the past to evaluate the prevalence of NPHPT in a different group of patients. According to this method, participants were identified as “normal” if they were inside the ellipse and “abnormal” if they were outside (Fig. 1). To get the different categories described below, researchers used the reference interval for adjusted calcium. However, they used the geometric mean of the reference interval for PTH (11). We have adjusted this method and brought it closer to everyday clinical practice by using the clinical reference intervals both for adjusted calcium and PTH. Doing so, patients were divided into 10 categories as described as follows. “High” or “low” refer to values of either adjusted calcium or PTH being greater than or less than the reference interval, respectively.

To compare the variability of different analytes in the 3 groups, the overall mean and the pooled within-subject SD of each analyte was calculated. The ANOVA method was used to calculate the within-subject variance because this method addresses the case of participants having different numbers of observations. To follow this method, we first checked the assumption that the SD was unrelated to the magnitude of the measurement. This was performed graphically, by plotting the individuals’ SDs against their means and analytically by calculating a rank correlation coefficient (12).

To compare characteristics between the groups at baseline, one-way ANOVA and Kruskal-Wallis were used to compare the quantitative data. The results are presented as mean and SD when ANOVA was used for the analysis, as geometric mean and 95% CI, when log transformation was performed before analysis with ANOVA, or as median and interquartile range if nonparametric tests were used for the analysis. The Mann-Whitney test was used to compare the 24-hour urine calcium between the NPHPT and PHPT groups. For the comparison of the overall measurements of PTH and adjusted calcium resulting from follow-up, we used a mixed linear model. Individual variability was modeled using a random effect and the difference between groups was estimated using a fixed effect.

The statistical analyses have been performed using R studio statistical software, version 1.1.442 (RStudio, Inc).

### Definitions of different groups

As mentioned in the “Statistics” section, patients were divided into 10 categories based on their laboratory intervals for adjusted calcium and PTH. The cutoff for normal vitamin D was 50 nmol/L according to the Royal Osteoporosis Society guidelines (13). The categories were defined as follows. Normal: anyone inside the ellipse; the rest of the groups described were outside the ellipse. Hyperparathyroid hypercalcemia (HH) included patients with PHPT, and familial hypocalciuric hypercalcemia (FHH) and was defined as anyone outside the ellipse having high adjusted calcium and high PTH. Hypoparathyroidism: low adjusted calcium and low PTH. Secondary hyperparathyroidism: a) low adjusted calcium and high PTH or, b) normal adjusted calcium and high PTH with 25(OH)D less than 50 nmol/L or eGFR less than 60 mL/min/1.73 m^2^. Non-PTH hypercalcemia: high adjusted calcium and low PTH. NPHPT: normal adjusted calcium and high PTH, given that 25(OH)D ≥ 50 nmol/L and eGFR ≥ 60 ml/min/1.73 m^2^. Normocalcemic hypoparathyroidism: normal adjusted calcium and low PTH. Normoparathyroid hypercalcemia: high adjusted calcium and normal PTH. Normoparathyroid hypocalcemia: low adjusted calcium and normal PTH. Unidentified: normal adjusted calcium and normal PTH but being outside the ellipse. The groups of interest for this study were the normal population and the patients with HH and NPHPT. To characterize these groups further, the following procedures were followed.

For the NPHPT patients identified from the index date, a careful evaluation of their medical records and other laboratory investigations were performed to check for exclusion criteria according to the guidelines (see the introductory section). Renal calcium loss was defined as having 24-hour urine calcium greater than or equal to 6.25 mmol/24 h for women or 7.5 mmol/24 h for men and/or 0.1 mmol/kg/24 h or greater (14). Moreover, and to be in line with the international guidelines on persistence, only patients with persistently normal adjusted calcium and elevated PTH on 2 consecutive occasions were included.

For the HH patients, only the patients with vitamin D greater than or equal to 50 nmol/L and eGFR greater than or equal to 60 mL/min/1.73m^2^ on the index date were chosen to be used for the variability analysis. An evaluation of their medical files excluded patients with other diseases such as renal transplant. Results after parathyroid surgery were excluded. Once again, only patients with consistently high calcium and PTH on at least 2 consecutive occasions were included. To include only patients with PHPT in this group, one or more of the following criteria were used: 24-hour urine calcium greater than 2.5 mmol/24 h or fractional excretion greater than 0.02; or surgically proven PHPT (correction of high calcium after parathyroid surgery). This was performed to exclude FHH. At this point, all remaining patients in this group were defined as PHPT.

For the control population, a random sample of 300 individuals from inside the ellipse having normal eGFR and being vitamin D replete (as defined previously) on the index date was chosen.

## Results

In total, 6293 patients attended the Metabolic Bone Centre and were assessed for secondary osteoporosis from January 2013 to July 2017. All these patients had a BMD measurement and a laboratory evaluation. Thirteen patients did not have PTH available on the index day and were excluded. The total number of patients analyzed was 6280; their mean age was 66 years (range, 16-100 years), and 4527 (72%) were female. After applying the Mahalanobis distance, the ellipse seen in Fig. 1 was drawn. In total, 5574 patients were inside the ellipse and were considered “normal,” whereas the rest were outliers and were divided into disease categories using the laboratory reference interval for calcium and PTH (Table 1).

**Table 1.** Different patient categories based on calcium metabolism disorders

Categories	No. (%)
Normal	5574 (88.76)
HH	172 (2.74)
Hypoparathyroidism	1 (0.02)
Secondary hyperparathyroidism	291 (4.63)
Non-PTH hypercalcemia	6 (0.10)
NPHPT	28 (0.45)
NHYPO	22 (0.35)
Normoparathyroid hypercalcemia	67 (1.07)
Normoparathyroid hypocalcemia	43 (0.68)
Unclassified abnormal	76 (1.21)

Normal: anyone inside the ellipse; the rest of the groups described were outside the ellipse. HH: high adjusted calcium and high PTH. Hypoparathyroidism: low adjusted calcium and low PTH. Secondary hyperparathyroidism: a) low adjusted calcium and high PTH or, b) normal adjusted calcium and high PTH with 25(OH)D < 50 nmol/L or eGFR < 60 ml/min/1.73 m^2^. Non-PTH hypercalcemia: high adjusted calcium and low PTH. NPHPT: normal adjusted calcium and high PTH, given that 25(OH)D ≥ 50 nmol/L and eGFR ≥ 60 ml/min/1.73m^2^. NHYPO: normal adjusted calcium and low PTH. Normoparathyroid hypercalcemia: high adjusted calcium and normal PTH. Normoparathyroid hypocalcemia: low adjusted calcium and normal PTH. Unclassified abnormal: normal adjusted calcium and normal PTH but outside the ellipse.

Abbreviations: 25(OH)D, 25-hydroxyvitamin D; eGFR, estimated glomerular filtration rate; HH, hyperparathyroid hypercalcemia; NHYPO, normocalcemic hypoparathyroidism; NPHPT, normocalcemic hyperparathyroidism; PTH, parathyroid hormone.

In total, 28 patients initially fulfilled the inclusion criteria for NPHPT (normal calcium, high PTH, both on at least 2 occasions, normal eGFR and vitamin D replete on index day). A careful evaluation of their medical records and their medical history excluded 17 patients (Table 2). The natural history was then studied, using calcium measurements available from January 2013 to the end of July 2018. Two patients were excluded because of PTH inconsistency (S2406 and S3820). Eleven patients were identified as having NPHPT with persistent confirmed results of normal calcium and elevated PTH on at least 2 occasions. The prevalence was 0.18% (95% CI 0.10-0.31). The mean age of these patients was 69 years, and 91% were female. None had parathyroid surgery.

**Table 2.** Patients checked for inclusion in normocalcemic hyperparathyroidism group

Study No.	Age, y	Sex	Medical Files Check	Persistence of Calcium and PTH	Natural History of Adjusted Calcium
S0189	65	F	Excluded—anticonvulsants		
S0227	59	F	Included	Yes	Persistent normocalcemia
S0449	65	F	Excluded—treated pseudohypoparathyroidism		
S0567	72	F	Excluded—tertiary hyperparathyroidism-renal transplant		
S0696	75	F	Excluded—bisphosphonates		
S0757	68	F	Included	Yes	Intermittent hypercalcemia
S0871	83	M	Excluded—bisphosphonates		
S0911	70	F	Included	Yes	Persistent normocalcemia
S1194	74	F	Excluded—bisphosphonates		
S1620	75	F	Included	Yes	Persistent normocalcemia
S1692	79	F	Excluded—bisphosphonates		
S1753	86	M	Included	Yes	Intermittent hypercalcemia
S2406	67	F	Included	No	
S2453	57	F	Included	Yes	Intermittent hypercalcemia
S2654	88	F	Excluded—Crohn— bisphosphonates—furosemide		
S2720	83	F	Included	Yes	Intermittent hypercalcemia
S3021	64	F	Included	Yes	Persistent normocalcemia
S3703	72	F	Excluded—anticonvulsants		
S3812	70	F	Included		Intermittent hypercalcemia
S3820	49	F	Included	No	
S3841	84	F	Excluded—bisphosphonates		
S3882	69	F	Excluded—hypercalciuria		
S4392	66	F	Included	Yes	Intermittent hypercalcemia
S4618	59	F	Included	Yes	Intermittent hypercalcemia
S4903	59	M	Excluded—renal transplant		
S5321	78	F	Excluded—bisphosphonates		
S5369	64	F	Excluded—hypercalciuria		
S5408	82	F	Excluded—furosemide		

Abbreviations: F, female; M, male; PTH, parathyroid hormone.

**Figure 2.**
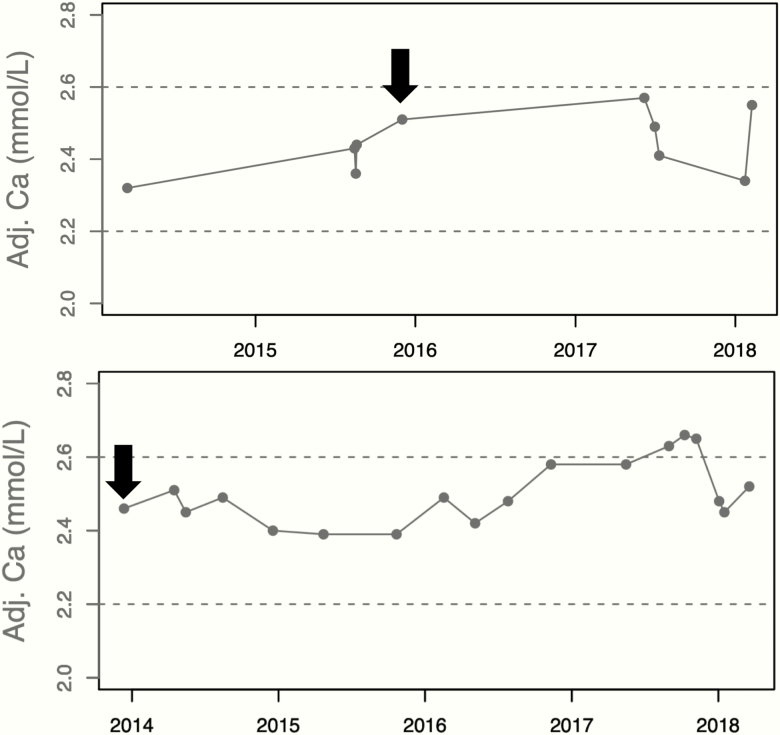
The 2 patterns identified in patients with normocalcemic hyperparathyroidism (NPHPT) when studying the natural history of adjusted calcium. Top figure, patient S1620, Persistent normocalcemia and, bottom figure, patient S2720, intermittent hypercalcemia. Arrows represent index day (day of bone mineral density scan). X-axes represent year of follow-up. Adj.Ca, adjusted calcium.

**Figure 3.**
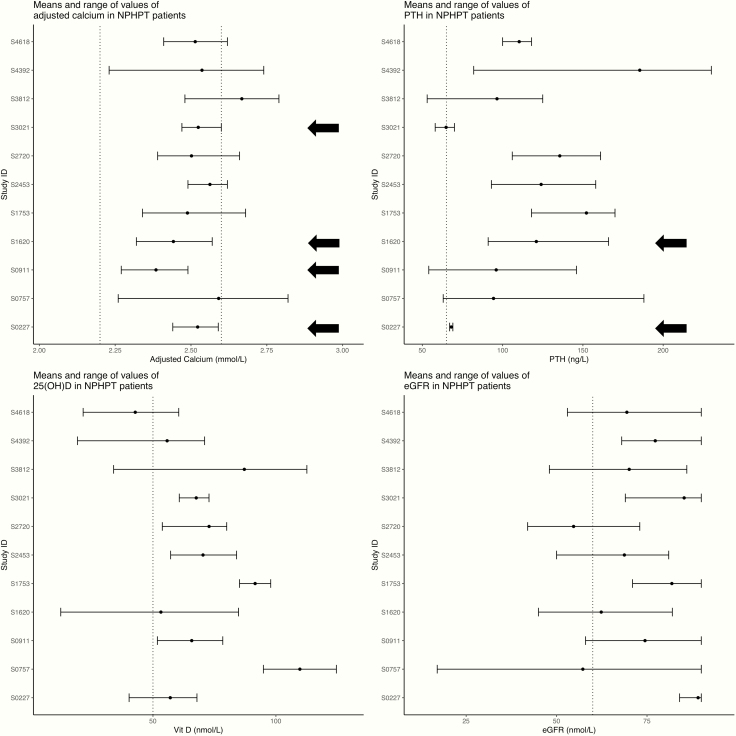
Means and range of values of different analytes in the 11 normocalcemic hyperparathyroidism (NPHPT) patients. The dashed lines represent the reference interval for adjusted calcium, the upper reference interval for parathyroid hormone (PTH), and the desired level for 25(OH)D and estimated glomerular filtration rate (eGFR). Persistent normocalcemia was rare, only occurred in 4 patients (shown in the graph with arrows). However, only 2 patients (S0227 and S1620) had consistently high PTH. These patients were not consistently vitamin D replete and/or did not have consistently normal eGFR. PHPT, primary hyperparathyroidism.

Two patterns were identified while studying the natural history: persistent normocalcemia and intermittent hypercalcemia). Intermittent hypercalcemia occurred in 7 patients (Fig. 2, lower panel). Persistent normocalcemia was rare and occurred in only 4 patients (0.06% of the whole population) (Fig. 2, upper panel). However, only 2 of these patients (S0227 and S1620) had consistently high PTH, but they were not consistently vitamin D replete and/or did not have consistently normal eGFR (Fig. 3). If the international guidelines were strictly applied, the prevalence of NPHPT in this population would be zero.

Out of the 172 patients identified with HH, 29 were vitamin D replete and had normal eGFR on the index date. The evaluation of their medical records excluded one patient because of previous renal transplant (S2706). One patient had his or her index date after parathyroid surgery and was also excluded (S5826). Eight patients were excluded because they had one only result for PTH, and the persistence could not be confirmed, and one patient was excluded for not having at least 2 consistently high calcium measurements. One patient was excluded because FHH could not be ruled out because of lack of urine calcium (S2031). The final PHPT group had 17 patients (mean age, 69; 89% female). Twelve patients had persistent hypercalcemia (70%), whereas the remaining 6 had intermittent hypercalcemia, a pattern also seen in patients with NPHPT.

The control population group consisted of 300 individuals (mean age, 67 years; 71% female). There was no statistically significant difference in age or sex distribution between the groups (all *P* > .05).

The baseline characteristics on the index date are summarized in Table 3. The 3 groups had significant differences in adjusted calcium on the index date. PTH did not differ between the NPHPT and PHPT groups. The groups had similar age- and sex-adjusted BMD results. Phosphate was significantly lower in the PHPT group compared to the control group. All the other variables studied did not have any differences between the 3 groups at baseline.

**Table 3.** Characteristics of the 3 groups on index date

	Control (n = 300)	NPHPT (n = 11)	PHPT (n = 17)	*P*
Female (%)	214 (71)	10 (92)	15 (88)	.122
Age, y^*a*^	70 (20)	68 (11)	67 (6)	.975
BMI (g/cm^2^)^*c*^	25.6 (25.0, 26.3)	30.1 (24.4, 34.0)	26.2 (23.4, 28.9)	.303
PTH (ng/L)^*c*^	42.5 (40.8, 44.2)	106.8 (86.9, 123.9)	102.4 (89.0, 112.4)	**<** **.001**
Adjusted calcium (mmol/L)^*b*^	2.37 (0.08)	2.55 (0.05)	2.75 (0.11)	**<** **.001**
Phosphate (mmol/L)^*b*^	1.12 (0.18)	1.04 (0.14)	0.89 (0.16)	**<** **.001**
Alkaline phosphatase (IU/L)^*a*^	78 (37)	98 (33)	88 (27)	.070
25(OH)D (nmol/L)^*a*^	78.9 (32.9)	62.8 (23.5)	71.4 (30.5)	.083
Z score spine^*b*^	–0.1 (1.7)	0.2 (2.2)	–0.2 (1.3)	.932
Z score neck^*b*^	–0.4 (1.0)	–0.1 (1.3)	–0.4 (0.8)	.770

Results of pairwise comparisons: PTH in the control group differed significantly from both the NPHPT and PHPT groups, but there was no statistically significant difference between the NPHPT and PHPT groups. Adjusted calcium differed significantly between all groups. Phosphate differed significantly only between the PHPT and control group.

Abbreviations: 25(OH)D, 25-hydroxyvitamin D; BMI, body mass index; n, number of patients; NPHPT, normocalcemic hyperparathyroidism; PHPT: primary hyperparathyroidism; PTH, parathyroid hormone. Values in bold were significant.

^*a*^Shown as median (interquartile range).

^*b*^Shown as mean (SD).

^*c*^Shown as geometric mean (95% CI).

**Figure 4.**
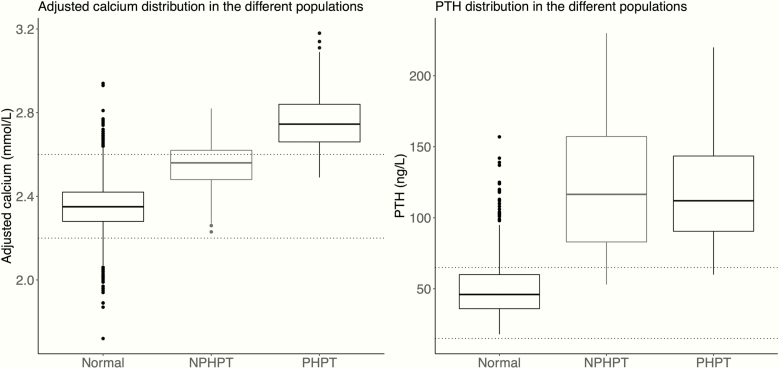
Boxplots showing the variability of calcium and parathyroid hormone (PTH) in the normal population and patients with primary hyperparathyroidism (PHPT) and normocalcemic hyperparathyroidism (NPHPT). The mean adjusted calcium was found to be significantly lower in the NPHPT group compared with the PHPT group (*P* < .001). The normal group had significantly lower adjusted calcium levels than all the other groups (*P* < .001). PTH did not differ significantly between these groups but was significantly higher than the normal population (*P* < .001).

After taking all the follow-up measurements into account, the mean adjusted calcium was still found to be significantly lower in the NPHPT group compared with the PHPT group (2.52 and 2.74 mmol/L, respectively). The control group had significantly lower adjusted calcium levels than all the other groups (2.35 mmol/L, all pairwise comparisons *P* < .001). PTH did not differ significantly between NPHPT and PHPT (115.4 and 116.0 ng/L, respectively) but was significantly higher than in the control group (48.1 ng/L, *P* < .001) (Fig. 4). The PHPT and NPHPT groups had similar calcium variability (within-subject SD [95% CI]: 0.088 [0.079, 0.097] and 0.089 [0.080, 1.000] mmol/L, respectively). The variability of calcium in the control group was slightly lower than for PHPT and NPHPT (within-subject SD 0.083 [0.080, 0.085] mmol/L). The 24-hour urine calcium was higher in the PHPT group compared to the NPHPT groups (mean, 7.51 and 4.25 mmol/24 h, respectively) but the difference was not significant (*P* = .063).

## Discussion

The international guidelines suggest that patients with NPHPT should have consistently normal values of calcium. Moreover, PTH should be high on at least 2 to 3 consecutive measurements. However, we observed in this population that persistent normocalcemia is rarely the case and using a single measurement to identify NPHPT patients can lead to overestimation of its prevalence. Patients with NPHPT usually have intermittent hypercalcemia as do PHPT patients, and we believe that NPHPT is a variant of PHPT. The intermittent nature of serum calcium both in NPHPT and PHPT results from the relatively high variability of serum calcium in all patient groups.

The studies available for the prevalence of NPHPT use different definitions and patient populations. Often the baseline data are the only ones analyzed, without evaluation of persistence. To study the natural history, only one further measurement during follow-up is used and the variability of calcium in the follow-up period is not described.

To our knowledge, only 2 studies have reported the prevalence of NPHPT from referral centers (Table 4), one of them having the highest prevalence in the literature (8.9%) (15). The mean adjusted calcium was similar between the NPHPT group and the rest of the patients, a pattern not seen in our study. They also reported higher levels of PTH in the NPHPT group (109.5 [45.2] vs 39.1 [14.3] pg/mL, *P* < .001] (15). Another estimate of prevalence from a referral center is from a group in the Czech Republic. They report 187 patients (1.2%) with NPHPT (16). The prevalence of NPHPT was also studied in the general population and was reported to be between 0.1% and 6% (Table 4).

**Table 4.** Studies evaluating prevalence of normocalcemic hyperparathyroidism

Study	Population	No. of NPHPT Patients (%); Mean Age, y;Sex	Definition of NPHPT	Comments/Limitations
Marques et al Brazil (referral center) (15)	Analyzed records of 156 postmenopausal women referred to hospital to be screened for osteoporosis	14 (8.9); 60.6; 100% female	At least 2 samples of adjusted Ca and PTH. Excluded: 25(OH)D < 30 ng/mL, GFR < 40 mL/min, medications (bisphosphonates, diuretics, anticonvulsants, lithium), metabolic bone diseases, GI diseases with malabsorption, liver disease, incomplete records	Lower cutoff for eGFR
Šiprová et al Czech Republic (referral center) (16)	15 343 referrals to endocrine center. PTH measured in 1180 (patients with pathological or marginal levels of total Ca, ionized Ca, serum phosphate, patients with reduced BMD, and with possible PHPT diagnosis from medical history)	At baseline: 187 (1.2); 61.1; 81% female; at follow-up: 151	Normal total and ionized Ca and high PTH at first visit. 25(OH)D ≥ 20 ng/ml (patients with low vitamin D were treated, and PTH had to be elevated after retest at 3 mo). Excluded cases with renal insufficiency, Ca malabsorption, hypercalciuria, medications (PPI, thiazides, lithium)	Not clear if they excluded people on bisphosphonates, GFR cutoff not given
Berger et al Canada (community) (17)	Population-based Canadian Multicentre Osteoporosis Study: prospective cohort of 9423 community-dwelling women and men living within 50 km of 9 Canadian cities. 566 men and 1306 women (n = 1875) age ≥ 35 y with available PTH	62 (3.31); NA; NA	Normal total Ca and high PTH, 25(OH) D ≥ 50 nmol/L, eGFR ≥ 60 mL/ min/1.73 m^2^	85% users of antiresorptives and diuretics. Not clear if they checked persistence or other causes of secondary hyperparathyroidism
Cusano et al US (community) (18)	DHS: population-based cohort study. Evaluated 3450 individuals age 18 to 65 y with Ca and PTH values.2122 patients had follow-up data 8 y later	At baseline: 108 (3.1); 41.3; 38% female; at follow-up: 13 (0.6%)	Normal albumin-adjusted Ca and high PTH. Excluded renal insufficiency (GFR < 60 mL/min), 25(OH)D ≤ 20 ng/ mL, thiazide, or lithium use	Only single laboratory values and did not check persistence.Lack of data regarding medical history and parathyroid surgery.Not clear if they excluded patients on bisphosphonates or with hypercalciuria
Cusano et al US (community) (18)	Osteoporotic Fractures in Men, an unselected community-based study in age ≥ 65 y. Evaluated 2364 men with calcium and PTH values	9 (0.4); 70.0; 0% female	Normal albumin-adjusted Ca and high PTH. Excluded renal insufficiency (GFR < 60 mL/min), 25(OH)D ≤ 20 ng/ mL, thiazide use	Same as DHS
García-Martín et al Spain (community) (19)	Prospective study of 100 healthy postmenopausal women. All had follow-up 6 y later	6 (6); 56.3; 100% female; at follow-up: 6	Normal adjusted Ca and high PTH. 25(OH)D > 30 ng/mL, normal renal function (creatinine clearance > 70 mL/ min/1.73 m^2^)	Not clear if they excluded patients on medications. Not clear what they defined as “healthy.”
Kontogeorgos et al Sweden (community) (20)	Random population sample of 2400 men and women age 25 to 64 years from World Health Organization MONItoring of trends and determinants for CArdiovascular disease project. Investigation in 1995, data on 608, including all women age 45 to 64 y, every fourth woman age 25 to 44 y, and every fourth man in all age groups (25-64 y), n = 410.	12 (2.0%); 53.3; NA; at follow-up: 1 (0.2%)	Normal total Ca and high PTH, 25(OH) ≥ 50 nmol/L, normal renal function	Patients on bisphosphonates and diuretics. Only one blood measurement at baseline
Lundgren et al Sweden (community) (21)	Population-based mammography screening in 5202 women age 55 to 75 years	28 (0.5); no data on age; 100% female	Normal ionized Ca. Creatinine < 160 μmol/L and either a) serum Ca < 2.50 mmol/L + PTH > 55 ng/L or b) serum Ca 2.50 to 2.60 mmol/L + PTH ≥ 35 ng/L. Checked for persistence (≥ 3 occasions). Excluded malabsorption and family history of hypercalcemia	Did not exclude patients on medications known to cause secondary hyperparathyroidism. No vitamin D check
Palermo et al Five European cities in UK, France, Germany (community) (11)	Recruited 2419 women (age 55-79 y) and 258 women (age 30-40 y) for Osteoporosis and Ultrasound Study. Follow-up after 6 y in 1416 patients	1 (0.1); no information on age; 100% female; at follow-up: none	Mahalanobis distance used: NPHPT anyone outside ellipse with normal adjusted Ca, high PTH, 25(OH) D ≥ 50 nmol/L, GFR ≥ 60 mL/min	Unclear if they excluded patients with other causes of secondary hyperparathyroidism (diseases, medication)
Rosário et al Brazil (community) (22)	Prospectively recruited adults ≥ 18 y to undergo thyroidectomy for nodular disease. Excluded patients who had ultrasound because of PHPT, patients with a history of nephrolithiasis, nephrocalcinosis, and pathological fracture, personal or family history of multiple endocrine neoplasia, or diagnosis of medullary thyroid cancer. N = 676	Criterion 1: 46 (6.8%). Only 8.7% had altered parathyroid glands (adenoma) during gland exploration (0.6% of cohort) Criterion 2: 30 (4.4%). Confirmed pathology: 13.3% Criterion 3: 12 (1.8%). Confirmed pathology: 33.3% Criterion 4: 5 (0.74%). Confirmed pathology: 80%	Criterion 1: Normal adjusted and ionized Ca and high PTH, confirmed at 2 measurements, 25(OH)D ≥ 20 ng/ dL, eGFR ≥ 40 mLl/min/1.73 m^2^. Excluded: those on diuretics, lithium, bisphosphonates, denosumab, recombinant PTH, corticosteroids; patients with primary aldosteronism, suspicion or known diagnosis of malabsorption, hyperphosphatemia, Ca/urinary creatine ratio ≥ 0.25, or thyroid dysfunction. Screened for celiac disease and excluded patients with positive antibodies Criterion 2: same as criterion 1 but 25(OH)D ≥ 20 ng/dL, eGFR ≥ 60 mL/ min/1.73 m^2^ Criterion 3: same as criterion 1 but 25(OH)D ≥ 30 ng/dL, eGFR ≥ 40 mL/ min/1.73 m^2^ Criterion 4: same as criterion 1 but 25(OH)D ≥ 30 ng/dL, eGFR ≥ 60 mL/ min/1.73 m^2^	
Vignali et al Italy (community) (23)	Residents of village in Southern Italy in 2010 (685 with full data)	3 (0.4); 47; 0% female	Normal adjusted Ca and high PTH, 25(OD)D ≥ 30 ng/mL, eGFR ≥ 60 mL/ min/1.73 m^2^. Excluded people on bisphosphonates and thiazides, overt GI and metabolic bone diseases	Did not check persistence and could not check urine Ca

Abbreviations: 25(OH)D, 25-hydroxyvitamin D; BMD, bone mineral density; Ca, calcium; DHS, Dallas Heart Study; eGFR, estimated glomerular filtration rate; GI, gastrointestinal; NA, not available; NPHPT, normocalcemic hyperparathyroidism; PHPT: primary hyperparathyroidism; PPI, proton pump inhibitor; PTH, parathyroid hormone.

The results for the natural history remain controversial with some studies reporting no conversion to hypercalcemia (19, 24, 25), whereas others report a small percentage of patients becoming hypercalcemic (16, 18, 20, 26, 27). The problem with the studies on natural history is that, although they might check persistence at baseline on 2 to 3 occasions, they then check the laboratory measurements on only 1 occasion after a certain period; if at that point the patient has high calcium, they conclude that the patient has progressed to hypercalcemia. All the studies are summarized in Table 5. Silverberg and Bilezikian found hypercalcemia in 3 patients after 1 year of follow-up. The researchers from this paper first proposed that NPHPT is the first phase of a biphasic disease course, which can be followed by hypercalcemic hyperparathyroidism (27). The group from the Czech Republic followed the 187 patients with NPHPT for 1 to 7 years. Out of them, 151 patients (81%) remained normocalcemic, and 36 (19%) became hypercalcemic; 24 (67%) increased their calcium to high levels within 2 years, 10 (28%) within 2 to 4 years, and 2 (5%) after more than 4 years. They also reported that 23 out of the 36 patients (64%) had persistent hypercalcemia, whereas 13 (36%) had intermittent hypercalcemia, a pattern also observed in our study. The mean baseline calcium of patients who remained normocalcemic was significantly lower than the ones that became hypercalcemic (16). A more recent study evaluating NPHPT patients going through parathyroidectomy reported that 40.8% of these patients had episodes of hypercalcemia more than 1 year before surgery (28).

**Table 5.** Studies evaluating natural history of normocalcemic hyperparathyroidism

Study	Study Population; Duration of Follow-up	Definition of NPHPT	Progression to Hypercalcemia
Ayturk et al (24)	20; 18 mo (6-mo intervals)	Normal Ca and high PTH confirmed in at least 3 measurements. Excluded chronic renal or liver failure, vitamin D deficiency, secondary hyperparathyroidism, treatment with lithium. No treatments with thiazide and loop diuretics, phenytoin, lithium, glucocorticoids, or oral contraceptives during study	None progressed to hypercalcemia
Tordjman et al (25)	20; 4.1 ± 3.2 y	Normal total Ca and high PTH secondary hyperparathyroidism excluded (impaired renal function). Three patients had low vitamin D levels but correction did not alter PTH levels and did not unmask hypercalcemia. Six patients had > 300 mg/24 h urine Ca and were given thiazides without affecting PTH levels. Persistence not checked at baseline	None of the patients developed hypercalcemia. Mean serum calcium levels did not change significantly (baseline vs last)
Garcia-Martin et al (19)	6; 1 y	Normal adjusted Ca and high PTH. 25(OH)D > 30 ng/mL, normal renal function (creatinine clearance > 70 mL/min/1.73 m^2^). Persistence not checked at baseline	All patients remained normocalcemic
Cusano et al (18)	64; 8 y	Normal albumin-adjusted Ca and high PTH. Excluded renal insufficiency (GFR < 60 mL/min), 25(OH)D ≤ 20 ng/mL, thiazide or lithium use. Persistence not checked at baseline	Hypercalcemia: 1 (1.6%). Persistent normal Ca, high PTH: 13 (20%)
Diri et al (26)	16; 4 y	Normal total Ca and high PTH, 25(OH)D > 20 ng/mL, repeated Ca and PTH measurements 3× with 2-wk intervals, no history of renal or liver diseases, no prescriptions known to affect Ca level	One (6.25%) developed hypercalcemia
Kontogeorgos et al (20)	12; 13 y. First assessment 1995, second 2008 to 2009, participation rate 67%	Normal total Ca and high PTH, 25(OH) ≥ 50 nmol/L, normal renal function	One (8.33%) developed hypercalcemia. Persistent normal Ca, high PTH: 1 (8.33%). Two had vitamin D deficiency, normal Ca, and high PTH
Silverberg et al (27)	22; up to 1 y	Normal adjusted calcium and high PTH. Confirmed on at least 2 occasions, 8 patients had normal ionized Ca, 25(OH) D > 20 ng/mL. Excluded FHH, liver disease, renal disease, urinary calcium > 87.5mmol/24h, GI disease with malabsorption, metabolic bone disease, medications (lithium, thiazide, oestrogens, loop diuretics, bisphosphonates, anticonvulsants)	Three (14%) developed hypercalcemia
Siprova et al (16)	187; 1 to 7 y	Normal total and ionized Ca and high PTH. 25(OH)D ≥ 20 ng/ ml (patients with low vitamin D were treated, and PTH had to be elevated after retested at 3 mo). Excluded cases with renal insufficiency, calcium malabsorption, hypercalciuria, medications (PPI, thiazides, lithium)	151 (81%) remained normocalcemic for whole follow-up period. 36 (19%) became hypercalcemic
Lowe et al (29)	37; 3.1 ± 0.3 y	Normal adjusted Ca and high PTH, 25(OH)D ≥ 50 nmol/L Excluded cases with renal insufficiency (GFR < 40 mL/min/1.73 m^2^), liver disease; significant hypercalciuria > 350 mg/24 h, thiazide diuretic or lithium use, other metabolic bone diseases (eg, Paget disease)	Seven (19%) became hypercalcemic. Patients who became hypercalcemic had higher Ca levels, higher urinary calcium excretion, and were older

Abbreviations: 25(OH)D, 25-hydroxyvitamin D; Ca, calcium; eGFR, estimated glomerular filtration rate; FHH, familial hypocalciuric hypercalcemia; NA, not available; NPHPT, normocalcemic hyperparathyroidism; PHPT: primary hyperparathyroidism; PPI, proton pump inhibitor; PTH, parathyroid hormone.

We believe that NPHPT is a variant of PHPT. This issue has been recently addressed by the first European Society of Endocrinology Workshop (PARAT). In its consensus statement, it mentioned that the individual variation in total calcium is 4 times narrower than the population reference interval and the interindividual variability. To ensure that a person has normal calcium, we need to know his or her individual range of variability. There is a chance that individuals might have an elevated calcium in comparison with their individual range, but still be within the normal population reference interval for calcium. These patients should probably be characterized as having PHPT and not NPHPT and be followed up and treated accordingly. This is of course after excluding causes of secondary hyperparathyroidism (30).

None of our patients progressed to persistent hypercalcemia; the patterns observed were either persistent normocalcemia or intermittent hypercalcemia. The 4 patients with persistent normocalcemia did not fulfill all the other criteria according to the international guidelines. Our hypothesis is supported by the fact that the underlying pathology of NPHPT seems to be similar to PHPT. A few studies report that multiglandular disease is more common in NPHPT (31-34) but not all reach statistical significance (35-37). However, patients included in these studies probably had secondary hyperparathyroidism resulting from vitamin D deficiency and CKD (32, 34). Most studies report that the average adenoma weight was lower in the NPHPT group compared to the PHPT group of patients (31, 35, 38).

This hypothesis, however, is not fully supported by recently published genetic data on NPHPT. Based on the hypothesis that NPHPT can be caused by PTH resistance (38), and the fact that the single-nucleotide polymorphism rs17251221 (A986S) in the calcium sensing receptor has been linked to PTH resistance, researchers in Spain recently investigated the effect of this single-nucleotide polymorphism in NPHPT patients. They recruited and prospectively studied 61 consecutive patients with NPHPT and asymptomatic PHPT. These patients had a follow-up of 1 year to check for the persistence of their laboratory investigations on at least 2 occasions. There were 38 patients (n = 24 NPHPT and n = 14 PHPT) with the wild-type genotype A986A and 23 S allele carriers (n = 20 A986S or n = 3 S986S). Seventeen NPHPT patients were S allele carriers. In these patients, the S allele was associated with significantly higher levels of serum-intact PTH (*P* = .024) after adjusting for factors such as vitamin D and calcium concentrations. There was no association with the S allele in PHPT patients (39).

In our series, we found a similar variability of adjusted calcium in patients with NPHPT and PHPT. The variability of calcium and PTH has been studied previously in a prospective series of surgically proven PHPT. In this study (6), the definition of NPHPT was every measurement of calcium being less than 10.2 mg/dL (2.55 mmol/L), and only 1.1% of the patients fulfilled this criterion. Almost all the patients with PHPT had variable calcium levels, with 74% having intermittent hypercalcemia within the previous year. This study did not include a control group but mentioned that serum calcium was more variable in PHPT patients compared to the same patients before developing this disorder. The variability before was described to be 0.19 (0.09) mg/dL (0.0475 [0.0225] mmol/L). After conversion to PHPT, the variability more than doubled (0.1 [0.0825] mmol/L), with calcium varying by more than 1 mg/dL (0.25 mmol/L) from month to month in some cases. The authors suggested this could be a loss of calcium homeostasis by a parathyroid tumor. Patients with NPHPT were reported as an exception, presenting with minimal variability, like the one in the patients before PHPT. Minimal variability is not the finding from our study, because NPHPT and PHPT patients both had similar calcium variability (6).

The variability described in calcium in these patients complicates their monitoring. According to the international guidelines, calcium and PTH should be tested every year, whereas a BMD check should be performed every 1 to 2 years. If there is a progression to hypercalcemia, patients should be treated according to the guidelines on asymptomatic PHPT (40). However, a majority of these patients have intermittent hypercalcemia; the subsequent results might be confusing both for the care provider and the patient as to whether surgery would be recommended.

We used a statistical approach to define the different categories in this population. Our results showed that a large percentage of participants were considered “normal” (inside the ellipse) using this approach, even though they had elevated PTH according to the reference interval. The individuals who were normal but had higher PTH than the reference interval were found to be significantly older than those who were normal and also had normal PTH according to the current reference interval (data not shown). These results align well with the fact that PTH is known to increase with age. This increase was recently found to be independent of 25(OH)D, ionized calcium, phosphate, and renal function (41). It has been previously suggested that age-related PTH reference intervals should be established (2). Our study supports this statement. By using the current reference interval in everyday practice, we are probably overdiagnosing NPHPT and may be subjecting older individuals to unnecessary testing. An increase of the upper limit of the reference interval in PTH could alter the number of patients diagnosed with this disorder.

This study describes the largest population ever studied to identify NPHPT and provides data during a long follow-up period (5 years). There are several limitations to this study, however. The samples were not fasting, blood was taken with a tourniquet, and ionized calcium was not available. The methods for vitamin D and eGFR changed during the follow-up period and that might have affected some results. We have used a 25(OH)D threshold of 50 nmol/L for identifying high PTH due to D deficiency, but the cross-sectional data indicate that values between 50 and 75 nmol/L might be associated with slight elevations of PTH, thus resulting in overdiagnosis of NPHPT. Many researchers recommend using a higher cutoff of 75 nmol/L. We have used the cutoff advised by the Royal Osteoporosis Society guidelines and the Fourth International Workshop on Asymptomatic Primary Hyperparathyroidism (2, 13). The impact of implementing the CKD-EPI equation is that it may have improved the accuracy in patients with an eGFR of 50 to 60 mL/min/1.73 m^2^ and might have resulted in fewer people being clustered as CKD. However, it might have increased the number of older people having CKD. The equation used to calculate the pooled SD cannot really distinguish between variations due to the progression of the disease or those due to natural variation. We assume that when taking measurements close to each other, the pooled SD would estimate the method variation and the variability of the assay. Because the measurements in this population are further away, we believe that the variability is due to the within-subject variability. Moreover, none of our patients had progressive disease as seen in the natural history graphs, so we believe that this is all due to natural variation. This was a retrospective observational study and the number of measurements varied from patient to patient. The interval between the measurements was not the same, as expected in a real-life setting. This limited the possibility of further analyses. The analysis for the within-subject SD did not take into account the effect of time. Ideally, to give a more accurate estimate of the variance, a prospective observational study should be designed having the same number of measurements for each patient at regular intervals. The analysis should preferably be conducted using a model that takes time into account. Despite the limitations, this study provided a guide to how adjusted calcium varies between the different groups described.

The patients studied were identified from a referral center and were evaluated for causes of secondary osteoporosis so they might be different from the general population. On the other hand, NPHPT is usually diagnosed during the evaluation of secondary osteoporosis and thus, mainly found in referral centers. Studying a referral population has the advantage of evaluating how common NPHPT is in a referral population with osteoporosis and whether screening for NPHPT is useful.

Another group that is mentioned in this cohort, is the normoparathyroid hypercalcemia one. These patients were previously been described to progress to PHPT (42). It was not within the scope of this study to evaluate these patients further, but it is among our future plans.

**Figure 5.**
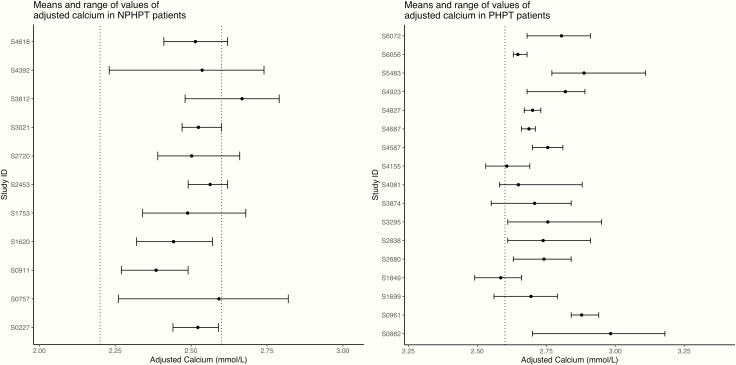
Means and range of values for adjusted calcium in the 11 normocalcemic hyperparathyroidism (NPHPT) patients and the 17 primary hyperparathyroidism (PHPT) patients. The dashed lines represent the reference interval. This figure shows that if the mean calcium was used to define the different groups, all but one patient in the left graph would be classified as NPHPT and all but one patient in the right graph would be classified as PHPT.

The question resulting from this study is whether NPHPT exists and, if it does, whether the current definition used is the most appropriate. We believe there are 3 ways forward. If we adjust the reference interval for PTH for age and use the current definition, NPHPT may not exist, as shown in this study. Another approach would be to revisit the current definition, and NPHPT should be defined as having the average calcium within normal limits (and not requiring it to be persistently normal). Using the same approach, PHPT should be considered as having a high average calcium. In this case, 10 out of our 11 included patients would be classified as NPHPT, and 16 out of 17 PHPT patients would be classified as having PHPT (Fig. 5).

Another way forward would be to consider NPHPT as a variant of PHPT and follow-up and treat according to the current guidelines as suggested in the consensus statement of the first PARAT. This is supported by many studies in the literature showing similar characteristics from these 2 groups of patients. It is also supported by the fact that the underlying pathology of NPHPT seems to be similar to PHPT. We believe, based on the results from this study, that this is the best approach to follow in clinical practice.

In conclusion, persistent normocalcemia in patients with NPHPT is rare. If the international guidelines were strictly applied, the prevalence of NPHPT in this population would be zero. Our study is the first that studies the natural history of patients with NPHPT in so much detail and provides data from a long follow-up period. We suggest that the definition be revisited and NPHPT be defined as having the average calcium within normal limits and not persistently normal, at least in a research setting. Subsequent hypercalcemia in these patients should be treated with caution, and any decisions for surgery should be made after persistently high levels of calcium have been confirmed. Another approach is to accept that NPHPT is a mild form of PHPT. Our recommendation for managing a patient with high PTH and normal calcium is continuous observation for the development of persistent hypercalcemic hyperparathyroidism.

## Abbreviations

BMD: bone mineral density
CI: Confidence interval
CKD: Chronic kidney disease
eGFR: estimated glomerular filtration rate
FHH: Familial hypocalciuric hypercalcemia
HH: Hyperparathyroid hypercalcemia
LSC: Least significant change
MBC: Metabolic Bone Centre
MD: Mahalanobis distance
NHYPO: Normocalcemic hypoparathyroidism
NPHPT: normocalcemic hyperparathyroidism
PHPT: primary hyperparathyroidism
PTH: parathyroid hormone
SD: Standard deviation
STH NHS FT: Sheffield Teaching Hospitals National Health Service Foundation Trust

## Additional Information

***Disclosure Summary:*** M.S. received funding for her fellowship from the Medical Research Council Centre of Excellence for Musculoskeletal Ageing and from Osteoporosis 2000 support group, and grant funding from Roche diagnostics. R.M.J., E.O., and N.F.A.P. have nothing to disclose. J.S.W. has received speaker honoraria from Eli Lilly and Sandoz; grant funding from Alexion and Immunodiagnostic Systems; donation of drug from Eli Lilly; Prostrakan (Kyowa Kirin) and Consilient for clinical studies; donation of assay kits from Biomedica; and consulting fees from Shire, Mereo Biopharma, Kyowa Kirin, UCB Pharma, and PharmaCosmos. R.E. receives consultancy funding from IDS, Roche Diagnostics, GSK Nutrition, FNIH, Mereo, Lilly, Sandoz, Nittobo, Abbvie, Samsung, and Haoma Medica; and grant funding from Nittobo, IDS, Roche, Amgen, and Alexion.

***Data Availability:*** The datasets generated during and/or analyzed during the current study are not publicly available but are available from the corresponding author on reasonable request.
